# Post-transplant cyclophosphamide in haploidentical stem cell transplantation: evaluating the impact of transplant conditioning intensity

**DOI:** 10.3389/fimmu.2025.1610391

**Published:** 2025-08-15

**Authors:** Umberto Pizzano, Simona Piemontese, Gabriele Facchin, Raffaella Greco, Marta Lisa Battista, Jacopo Peccatori, Renato Fanin, Fabio Ciceri, Francesca Patriarca, Maria Teresa Lupo-Stanghellini

**Affiliations:** ^1^ Haematology and Stem Cell Transplantation Unit, University Hospital ASUFC, Udine, Italy; ^2^ Haematology and Bone Marrow Transplantation Unit, IRCCS San Raffaele Scientific Institute, Milan, Italy; ^3^ Haematology and Bone Marrow Transplantation Unit, Cà Foncello Hospital, Treviso, Italy; ^4^ Department of Medical Area (DAME), University of Udine, Italy; ^5^ Università Vita-Salute San Raffaele, Milan, Italy

**Keywords:** haplo identical hematopoietic stem cell transplantation, PTCy-haplo HSCT, sirolimus, GRFS, transplant conditioning intensity, GvHD

## Abstract

**Background:**

The introduction of posttransplant cyclophosphamide (PTCy) is one of the major achievements in the field of haploidentical stem cell transplantation (haplo-HCT). The transplant conditioning intensity (TCI) score is a refined classification of conditioning regimens that assigns weight scores to conditioning regimen components. The aim of our analysis was twofold: to assess the effect on transplant outcomes of combining PTCy with calcineurin inhibitor + mycophenolate mofetil (MMF) instead of mTOR inhibitor + MMF for GvHD prophylaxis, and to assess the effect of stratification by conditioning intensity in the setting of haplo-HCT.

**Methods:**

This study was conducted in adult patients who underwent haplo-HCT at the University Hospital of Udine (UUH) or Ospedale San Raffaele (OSR) between January 2014 and December 2021. Patients received PTCy plus CsA-MMF at UUH and sirolimus-MMF at OSR. Conditioning intensity was defined by the TCI score. All data were collected prospectively.

**Results:**

A total of 216 haplo-HCTs were performed, 81 at UUH and 135 at OSR. Notably, none of the patients at UUH received a high TCI score regimen compared to 72 (53.3%) at OSR. Our results show overlapping survival outcomes (OS, NRM, DFS, GRFS, and RI) within the two platforms. We observed a higher incidence of cGvHD within the sirolimus/MMF + PTCy platform, and high TCI was found to be the only risk factor for a higher incidence of grades III–IV aGvHD in univariate analysis.

**Conclusion:**

Our results suggest that TCI may reveal the role of chemoradiotherapy in promoting conditions that may contribute to the occurrence of GvHD. The impact of moderate/severe cGvHD on quality of life must challenge our efforts to further optimise prophylactic strategies.

## Highlights

- CNI/MMF + PTCy vs. sirolimus/MMF + PTCy provided superimposable survival outcomes.
- Transplant conditioning intensity—a modifiable factor—may reveal the conditional contribution of conditioning to the occurrence of aGvHD.
- Sirolimus/MMF + PTCy is associated with an increased chronic GvHD incidence.

## Introduction

Advances in conditioning, graft-versus-host disease (GvHD) prophylaxis, and antimicrobial prophylaxis have improved the safety of allogeneic haematopoietic stem cell transplantation (HCT), leading to a substantial increase in the number of patients transplanted each year (1).

One of the major achievements in the field is represented by the widespread adoption of post-transplantation cyclophosphamide (2) (PTCy), which has increased the use of HLA-haploidentical HCT (haplo-HCT), greatly expanding the donor pool and paving the way toward the development of a new and effective strategy to prevent GvHD, also in the setting of matched related and unrelated transplants (3). In the attempt to identify the better combination of immunosuppressive drugs with PTCy, several groups have explored alternative strategies to the standard association with a calcineurin inhibitor (CNI) plus mycophenolate mofetil (MMF), introducing sirolimus (± MMF) and single-agent tacrolimus. A recent multicentre Spanish (4) study unveils the absence of differences in overall survival (OS), progression-free survival (PFS), nonrelapse mortality (NRM), or relapse incidence (RI) among three cohorts of patients treated with PTCy + single-agent tacrolimus, CNI + MMF, or sirolimus + MMF. Similarly, a phase 2 study from the Lee Moffitt Cancer Center (5) confirms superimposable results after haplo-HCT with CNI + MMF or sirolimus + MMF in combination with PTCy.

While the use of PTCy has homogenised the platform for GvHD prophylaxis in the haploidentical setting, there is still no common standard for optimal conditioning and its intensity. A refined conditioning regimen classification was recently proposed by the EBMT, assigning intensity weight scores for frequently used conditioning regimen components in relation to their prognostic value for NRM, and using their sum to generate a transplant conditioning intensity (TCI) score (6, 7). The intention of this score was to try to standardise the intensity of a conditioning regimen more effectively than the existing nomenclature. Novel drugs (e.g., thiotepa) and optimised forms of drugs (e.g., treosulfan) with reduced nonhaematological toxicity are frequently used nowadays, and their different toxicity profiles are not considered in the current RIC/MAC classification scheme (6).

The aim of our real-life analysis was twofold: first, to assess the effect on transplant outcomes of combining PTCy with calcineurin inhibitor + MMF, rather than mTOR inhibitor + MMF, for GvHD prophylaxis; and second, to evaluate the effect of stratification by conditioning intensity in the setting of haplo-HCT.

## Patients and methods

This is an observational retrospective study. All consecutive adult patients who received a haplo-HCT between January 2014 and December 2021 in two Italian centres, Udine University Hospital (UUH) in Udine and Ospedale San Raffaele (OSR) in Milan, were included in the analysis. All patients received PTCy plus CsA-MMF at UUH and sirolimus-MMF (8) at OSR.

Data of patients, transplant procedures, and complications were prospectively collected in all cases and then entered into a computerised database. Clinical charts were additionally reviewed for inconsistent or missing data.

The primary endpoint of the study was the incidence of GvHD-free, relapse-free survival (9). Secondary outcomes were OS and NRM. The last follow-up was on 14 July 2023. OS was defined as the time from transplant to death from all causes. Disease-free survival (DFS) was defined as the time to death or relapse/progression, whichever occurred first. GvHD-free/disease-free survival (GRFS) was defined as survival without grades III–IV acute GvHD (aGvHD), moderate-to-severe chronic GvHD (cGvHD), relapse, or death. Myeloid engraftment was defined as the first of three consecutive days with neutrophil counts ≥ 0.5 × 10^9^/L after transplantation. Platelet engraftment was defined as the first of seven consecutive days with platelet counts ≥ 20 × 10^9^/L without platelet transfusions. Clinical diagnosis and grading of aGvHD and cGvHD were made according to the MAGIC criteria (10) and the National Institutes of Health consensus criteria (11), respectively. For aGvHD and cGvHD, death and relapse were considered competing events.

### Statistical analysis

Patient and transplantation characteristics for each cohort were compared using the Chi-square test for categorical variables and the Mann–Whitney *U* test for continuous variables. Overall survival, DFS, and GRFS were estimated using the Kaplan–Meier product-limit method (12). Cumulative incidence (CI) functions (13) were used to estimate engraftment, aGvHD, cGvHD, NRM, and RI. Univariate analyses were performed using the log-rank test (14) for OS, DFS, and GRFS, while Gray’s test (15) was used for CI outcomes. The following variables were assessed via univariate analysis: type of transplant centre (OSR vs. UUH), patient age (≤ or > 57 years, median value), donor age (≤ or > 38 years, median value), female donors for male patients (vs. other combinations), host/donor CMV IgG status (neg/neg vs. other combinations), and intensity of conditioning regimen (TCI low, intermediate, high).

Multivariable analyses (MVA) were performed using the Cox proportional hazards model (16). All factors known to influence outcomes, as well as those with a *p*-value < 0.10 in univariate analysis, were initially included in the model. If two variables were found to be strongly associated with each other, only the one with the most significant *p*-value in univariate analysis was entered into the Cox model.

All *p*-values were two-sided with a type 1 error rate fixed at 0.05. Statistical analyses were performed with SPSS 26 (SPSS Inc., Chicago, IL, USA) and R 4.0.3 (R Development Core Team, Vienna, Austria).

## Results

Between January 2014 and December 2021, 216 consecutive adult patients received an unmanipulated peripheral blood stem cell haplo-HCT at UUH (81 patients) or OSR (135 patients) for haematological malignancies. The characteristics of the two patient cohorts are shown in **Table 1**.

**Table 1:** Patients characteristics.

	Overall	OSR	UUH	P
**Patient age (mean)**	57 (18-76)	57 (19-76)	59 (18-74)	0.854
**Median follow-up (months)**	29.5	37.8	21.1	***0.000024***
**Donor age (mean)**	38 (18-70)	37 (18-70)	39 (19-68)	0.916
**Female into male**	51 (23.6%)	31 (23%)	20 (24.7%)	0.448
**TCI score ** - Low • TBF • Treo-FLU • CY-FLU-TBI • Others - Intermediate • Treo-FLU • Others - High • TTF • Treo-Flu-Mel • Treo-Flu-TBI	65 (30%) 38 (17.6%) 10 (4.6%) 11 (5%) 6 (2.8%) 79 (36.5%) 41 (19%) 38 (17.5%) 72 (33.5)% 55 (25.5%) 12 (5.5%) 5 (2.5%)	6 (4.4%) 57 (42.2%) 72 (53.3%)	59 (72.8%) 22 (27.2%) 0 (0%)	***0.00706x10^-25^***
**Diagnosis** - Acute Leukemia - Other diseases • HL • MDS • NHL • MPN • MDS/MPN • MM	149 (70%) 67 (30%) 21 (9.7%) 18 (8.3%) 13 (6%) 9 (4.1%) 4 (1.9%) 2 (0.9%)	98 (72.6%) 37 (27.4%) 8 (5.9%) 13 (9.6%) 8 (5.9%) 5 (3.7%) 3 (2.2%) 0	51 (63%) 30 (37%) 13 (16%) 5 (6.1%) 5 (6.1%) 4 (4.9%) 1 (1.2%) 2 (2.4%)	0.092
**Disease status at transplant ** - Acute Leukemia not in CR - Others	61 (28.2%) 155 (71.8%)	36 (26.7%) 99 (73.3%)	25 (30.9%) 56 (69.1%)	0.535

TCI, transplant conditioning intensity; TBF, Thiotepa-Busulfan-Fludarabine; Treo-FLU, Treosulfan-Fludarabine; CY-FLU-TBI, Cyclofosphamyde-Fludarabine-TBI 2 Gy; TTF, Treosulfan-Thiotepa-Fludarabine; Mel, Melphalan; Treo-Flu-TBI, Treosulfan-Fludarabine-TBI 4 Gy; CR, complete remission; HL, Hodgkin Lymphoma; MDS, Myelodysplastic syndrome; NHL, non-Hodgkin Lymphoma; MPN, Myeloproliferative Neoplasms; MM, Multiple Myeloma.

Statistically significant variables/values are in bold/italics.

Patients in UUH and OSR were similar in terms of patients’ median age (59 years vs. 57 years, *p* = 0.854), diagnosis (acute leukaemia: 51 patients vs. 98 patients, *p* = 0.854), disease status (acute leukaemia not in remission: 25 patients vs. 36 patients, *p* = 0.535), donor median age (39 years vs. 37 years, *p* = 0.916), and donor/host sex combination (female/male patient: 20 vs. 31, *p* = 0.448). They differed in terms of median follow-up (21.1 months vs. 37.8 months, *p <*0.0001) and TCI (6, 7) (*p* < 0.0001). TCI score low regimens were performed in 59 patients (72.8%) at UUH and six patients (4.4%) at OSR. TCI score intermediate regimens were performed in 22 patients (27.2%) at UUH and 57 patients (42.2%) at OSR, while none of the patients at UUH received a TCI score high regimen, compared to 72 patients (53.3%) at OSR (*p* < 0.0001). Notably, patients at UUH mainly received a classic thiotepa + busulfan + fludarabine (TBF) conditioning regimen (60%) for both low and intermediate TCI, while a treosulfan-based conditioning regimen was selected for the majority of patients at OSR (98.5%). At OSR, treosulfan–fludarabine (Treo-FLU) was the most used intermediate TCI regimen, while treosulfan–thiotepa–fludarabine (TTF) and treosulfan–fludarabine–melphalan (Treo-Flu-Mel) were the most frequent regimens among high TCI. GvHD prophylaxis differs between the two centres, being based on cyclophosphamide (50 mg/kg on days + 3 and + 4 after transplant), MMF (10 mg/kg three times daily from days + 5 to + 28 after transplant), and sirolimus from day + 5 in OSR (8), while at UUH, ciclosporin (1.5 mg/kg twice a day from day + 5) was used instead of sirolimus.

The overall CI of neutrophil engraftment was 98% ± 1% at day 30. All patients showed full engraftment by day 100. The 3-year overall GRFS, OS, and DFS were as follows: 32% ± 3%, 57% ± 3%, and 52% ± 3%, respectively. Day 100 CI of aGvHD grades II–IV and III–IV were 31% ± 1% and 13% ± 5%, respectively. The 2-year CI of chronic GvHD overall and requiring systemic therapy was 36% ± 1% and 25% ± 1%, respectively. Day 100 and 3-year CI of NRM were 14% ± 1% and 25% ± 1%, respectively. The 3-year CI of RI was 23% ± 1%.

The univariate results according to transplant centre were as follows: 32% ± 6% at UUH vs. 32% ± 4% at OSR (*p* = 0.768) for GRFS, 51% ± 6% at UUH vs. 61% ± 4% at OSR (*p* = 0.273) for OS, 42% ± 6% at UUH vs. 58% ± 4% at OSR (*p* = 0.071) for DFS, 1% ± 1% at UUH vs. 20% ± 1% at OSR (*p* < 0.0001) for aGvHD grades III–IV, 15% ± 2% at UUH vs. 31% ± 2% at OSR (*p* = 0.007) for cGvHD requiring systemic therapy, 15% ± 1% at UUH vs. 13% ± 1% at OSR (*p* = 0.238) for day 100 NRM, and 28% ± 1% at UUH vs. 20% ± 1% at OSR (*p* = 0.285) for RI.

In univariate analysis, a higher TCI score was associated with a higher aGvHD grades III–IV CI (*p* < 0.0001): 28% ± 2% for TCI high, 9% ± 2% for intermediate, and 2% ± 1% for low TCI. It was also associated with a higher 2-year cGvHD moderate–severe CI: 29% ± 1% for high TCI, 23% ± 1% for intermediate, and 8% ± 1% for low (*p* = 0.014). Overall, 2-year OS, GRFS, NRM, RI, and overall cGvHD CI did not differ significantly according to TCI distribution. Conversely, TCI high showed a trend for a better 2-year DFS: 45% ± 6% for TCI low, 49% ± 6% for TCI intermediate, and 65% ± 6% for TCI high (*p* = 0.088). TCI high was found to be the only risk factor for a higher incidence of grades III–IV GvHD. We were not able to study the impact of GvHD prophylaxis on grades III–IV GvHD onset because of the predominant impact of TCI on this outcome and the strong association between TCI and GvHD prophylaxis.

The results of the MVA are shown in **Table 2**, while **Table 3** reports the results from the univariate analysis, limited to the risk factors identified in the MVA. Disease status at transplant is confirmed to have a crucial impact on major outcomes (OS, DFS, GRFS, and RI). Notably, older donors were associated with an increased risk of NRM (HR, 1.74 [CI 95%, 1–2.9]), any-grade and moderate/severe chronic GvHD (HR [2.05; CI 95%, 1.27–3.31] and HR, 2.21 [CI 95%, 1.26–3.86]), as well as lower GRFS (HR, 1.42 [CI 95%, 1.01–1.99]), while increased patient age negatively impacted only NRM (patient age ≥ 57 years: HR, 2.69 [CI 95%, 1.5–4.79]). Of note, in MVA, we observed a higher incidence of chronic GvHD (any-grade: HR, 0.47 [CI 95%, 0.27–0.83]; moderate/severe: HR, 0.4 [CI 95%, 0.2–0.8]) within the sirolimus/MMF + PTCy platform. The MVA showed no difference in OS, NRM, RI, DFS, aGvHD, and GRFS between the different GvHD prophylaxis platforms.

**Table 2:** Results of the multivariable analysis (MVA).

	*p*-value	HR	95% CI
OS			
Patient age > 57	0.169	1.336	0.88–2.01
Donor age > 38	0.195	1.312	0.87–1.98
***Acute leukaemia no CR vs. others***	***0.00001***	***2.566***	***1.69–3.9***
CMV nn	0.31	0.59	0.21–1.63
CNI/MMF vs. sirolimus/MMF	0.59	1.12	0.73–1.73
DFS			
Patient age > 57	0.071	1.43	0.97–2.12
Donor age > 38	0.159	1.32	0.9–1.95
***Acute leukaemia no CR vs. others***	***0.000003***	***2.59***	***1.73–3.86***
CNI/MMF vs. sirolimus/MMF	0.22	1.28	0.86–1.91
GRFS			
Patient age > 57	0.648	1.08	0.77–1.51
***Donor age > 38***	***0.04***	***1.42***	***1.01–1.99***
***Acute leukaemia no CR vs. others***	***0.004***	***1.70***	***1.19–2.44***
CMV nn	0.43	0.76	0.38–1.51
CNI/MMF vs. sirolimus/MMF	0.398	0.860	0.607–1.219
aGvHD 2–4			
Patient age > 57	0.958	0.988	0.623–1.566
Donor age > 38	0.071	1.534	0.964–2.443
CNI/MMF vs. sirolimus/MMF	0.953	1.014	0.630–1.634
cGvHD			
Patient age > 57	0.44	1.2	0.75–1.94
***Donor age > 38***	***0.003***	***2.05***	***1.27–3.31***
Acute leukaemia no CR vs. others	0.81	0.93	0.52–1.7
***CNI/MMF vs. sirolimus/MMF***	***0.009***	***0.474***	***0.27–0.83***
cGvHD ms			
Patient age > 57	0.93	0.97	0.56–1.7
***Donor age > 38***	***0.006***	***2.21***	***1.26–3.86***
***CNI/MMF vs. sirolimus/MMF***	***0.01***	***0.4***	***0.2–0.8***
NRM			
***Patient age > 57***	***0.001***	***2.69***	***1.5–4.79***
***Donor age > 38***	***0.044***	***1.74***	***1–2.9***
CNI/MMF vs. sirolimus/MMF	0.26	1.37	0.79–2.37
Relapse			
Patient age > 57	0.52	0.84	0.48–1.44
Donor age > 38	0.91	1.03	0.59–1.8
***Acute leukaemia no CR vs. others***	***1.88 × 10^−7^***	***4.29***	***2.48–7.43***
CNI/MMF vs. sirolimus/MMF	0.33	1.32	0.75–2.31

*aGvHD 2–4*, aGvHD grades II–IV; *cGvHD ms*, cGvHD moderate–severe; *CMV nn*, double-negative CMV serostatus; *CNI*, calcineurin inhibitor; *CR*, complete remission; *DFS*, disease-free survival; *GRFS*, GVHD-free relapse-free survival; *HR*, hazard ratio; *MMF*, mycophenolate mofetil; *NRM*, nonrelapse mortality; *OS*, overall survival; *RI*, relapse incidence.

Statistically significant variables/values are in bold/italics.

**Table 3:** Results from univariate analysis of risk factors included in the MVA.

	OS	DFS	GRFS	NRM	RI	aGvHD 2–4	cGvHD
Disease status							
Others	66% ± 4%	61% ± 4%	37% ± 4%	NA	16% ± 1%	NA	NA
Acute leukaemia no CR	34% ± 7%	27% ± 6%	17% ± 5%		43% ± 2%		
*p*-value	***0.000002***	***2.81 × 10^−7^***	***0.003***		***2.9 × 10^−6^***		
GvHD prophylaxis							
Sirolimus/MMF	N	NA	NA	NA	NA	NA	42 ± 2
CNI/MMF							23 ± 3
*p*-value							***0.007***
Patient age							
< 57	64% ± 5%	61% ± 5%	NA	5% ± 1%	NA	NA	NA
> 57	51% ± 5%	44% ± 5%		23% ± 1%			
*p*-value	***0.093***	***0.036***		***0.001***			
Donor age							
< 38	NA	NA	36% ± 5%	13% ± 1%	NA	NA	29% ± 2%
> 38			26% ± 5%	15% ± 1%			43% ± 3%
*p*-value			***0.064***	***0.098***			***0.03***

*aGvHD 2–4*, aGvHD grades II–IV; *cGvHD ms*, cGvHD moderate–severe; *CMV nn*, double-negative CMV serostatus; *CNI*, calcineurin inhibitor; *CR*, complete remission; *DFS*, disease-free survival; *GRFS*, GVHD-free relapse-free survival; *HR*, hazard ratio; *MMF*, mycophenolate mofetil; *NRM*, nonrelapse mortality; *OS*, overall survival; *RI*, relapse incidence.

Statistically significant variables/values are in bold/italics.

## Discussion

GvHD prophylaxis based on PTCy has revolutionised the haploidentical transplantation strategy over the past two decades due to its simplicity and efficacy. Currently, the focus is on achieving further improvements, such as optimising the pharmacological combination of GvHD prophylaxis with PTCy or adjusting the intensity of conditioning regimens.

The outcome data recorded in our patient cohort can be superimposed on the registry (17) data reported in contemporary cohorts, with a 2-year OS of 62.5% (95% CI, 60.6–64.4), 2-year NRM of 22.3% (95% CI, 20.7–23.9), 2-year DFS of 56.7% (95% CI, 54.8–58.6), 2-year cGvHD of 31.4% (95% CI, 29.6–33.3), and 6-month aGvHD grades III–IV of 9.6% (95% CI, 8.5–10.7). Similarly, our results are in line with those reported by both the Spanish (4) and American (5) groups, who compared different combinations (CNI- or sirolimus-based) with PTCy for the prevention of GvHD in the haploidentical setting.

Two observations, in particular, deserve further consideration. First, we sought to investigate the role of conditioning intensity as a potential contributor to overall outcomes. To our knowledge, this subanalysis in a haploidentical/PTCy-based context represents one of the first reported experiences. We assumed that TCI-based conditioning stratification could provide deeper insight into the impact of conditioning regimens on clinical outcomes, not only in an AML-restricted population. Our analysis showed that conditioning intensity did not significantly impact key transplant outcomes: 2-year OS, GRFS, NRM, RI, and overall cGvHD CI did not differ significantly according to TCI distribution. It is also worth noting a tendency for DFS to improve over time, although this did not reach statistical significance.

Regarding the effect on GvHD, we observed an association between high TCI and increased rates of moderate–severe cGvHD as well as higher grades III–IV aGvHD. The unequal distribution of TCI between the two GvHD prophylaxis cohorts—particularly the absence of high TCI conditioning in the CNI-based cohort—limited the multivariate analysis. Nevertheless, it is worth highlighting that, in addition to the prophylaxis platform, the role of conditioning intensity in influencing transplant outcomes, especially GvHD-related aspects, should be taken into account. We acknowledge the limitations of our case series and the inhomogeneous distribution, which prevented us from optimising the subanalysis. Nevertheless, these early data indicate that this variable cannot be overlooked in future evaluations. Further investigations and studies in larger cohorts will be crucial to clarify this issue.

We point out that the low day-100 incidence of grades III–IV aGvHD in the UUH cohort is consistent with reports from the Baltimore group in the context of nonmyeloablative, related haploidentical peripheral blood stem cell transplantation (18, 19). It is particularly interesting to observe that high TCI—in this study—was not associated with worse outcomes in terms of TRM and OS, despite the increased incidence of grades III and IV acute GvHD and moderate/severe chronic GvHD. Our hypothesis is that high TCI conditions are associated with a worse inflammatory profile, which may therefore increase the risk of GvHD. Improvements in therapeutic strategies for GvHD over time—such as the use of ruxolitinib for both acute and chronic GvHD, faecal transplants for acute GvHD, ibrutinib and belumosudil for chronic GvHD, and the availability of trials for new investigational drugs—have substantially improved survival outcomes. Similarly, the optimisation of supportive strategies has contributed to improved survival outcomes.

Secondly, the different GvHD prophylaxis platforms at the two centres must be acknowledged as a contributing factor (with an imbalance in the OSR cohort wherein high TCI conditioning was associated with sirolimus prophylaxis).

The finding of a trend toward a higher incidence of cGvHD with the sirolimus-based platform confirms the observations of the Spanish (3) and American (4) studies.

In our case series, we observed a 2-year incidence of cGvHD requiring systemic treatment (moderate/severe) of 25% ± 1%. Specifically, when comparing the CNI- and sirolimus-based regimens, the incidence was 15% ± 2% vs. 31% ± 2% (*p* = 0.007). The association with an increased incidence of cGvHD in the sirolimus-based platform reported in the univariate analysis was also confirmed in the multivariate analysis after adjustment for other risk factors. Similarly, the US study reported an incidence of moderate-to-severe chronic GvHD of 15% in the CNI-based cohort vs. 28% in the sirolimus-based cohort (*p* = 0.05), although this was not confirmed by multivariate analysis. The Spanish study again showed a lower incidence of moderate–severe cGvHD in the CNI-only arm compared to the CNI + MMF or sirolimus + MMF arm. This observation deserves attention, as it highlights a possible room for improvement in a clinical condition that can significantly impact patients’ quality of life. In conclusion, the multivariate analysis shows that, when comparing the two platforms associated with PTCy to prevent GvHD in haploidentical transplantation, the sirolimus-based platform is associated with a higher incidence of cGvHD, despite similar survival outcomes.

Of note, our study confirmed the role of disease status and donor/patient age as critical variables that may influence overall outcome (7). Disease remission at transplantation remains a key factor in all major posttransplant survival outcomes. Our case series also confirms the critical role of donor age, with a clear association between older donor age and higher NRM and worse GRFS.

Our present results show that TCI could be an effective tool to optimise the personalisation of the transplantation strategy and deserve further investigation and confirmation. Survival appears to be essentially superimposable with the two platforms—CNI/MMF + PTCy and sirolimus/MMF + PTCy—providing overlapping survival outcomes (OS, NRM, DFS, GRFS, RI). Considering the survival outcome, the impact on quality of life from moderate/severe cGvHD must challenge our efforts to further optimise prophylactic strategies. It will be imperative to further investigate the biological and functional aspects that can guide us in this challenge.

## Data Availability

The raw data supporting the conclusions of this article will be made available by the authors, without undue reservation.
